# Outcomes of Implementing a Multidimensional Antimicrobial Stewardship Program in a Medical Ward in a Third-Level University Hospital in Northern Italy

**DOI:** 10.3390/antibiotics14070683

**Published:** 2025-07-05

**Authors:** Maria Mazzitelli, Daniele Mengato, Gianmaria Barbato, Sara Lo Menzo, Fabio Dalla Valle, Margherita Boschetto, Paola Stano, Cristina Contessa, Daniele Donà, Vincenzo Scaglione, Giacomo Berti, Elisabetta Mariavittoria Giunco, Tiziano Martello, Francesca Venturini, Ignazio Castagliuolo, Michele Tessarin, Paolo Simioni, Annamaria Cattelan

**Affiliations:** 1 Infectious and Tropical Diseases Unit, Padua University Hospital, Via Giustiniani, 3, 35128 Padua, Italy; 2 Hospital Pharmacy Unit, Azienda Ospedale-Università Padova, 35128 Padua, Italy; daniele.mengato@aopd.veneto.it (D.M.); elisabettamariavittoria.giunco@aopd.veneto.it (E.M.G.);; 3 Haemorrhagic and Thrombotic Diseases Unit, Department of Medicine (DIMED), Padua University Hospital, 35128 Padua, Italy; 4 Medical Management Unit, Azienda Ospedale-Università Padova, 35128 Padua, Italy; 5 Microbiology and Virology Unit, Azienda Ospedale-Università Padova, 35128 Padua, Italy; 6 Department for Women’s and Children’s Health, Division of Pediatric Infectious Diseases, Padua University Hospital, 35128 Padova, Italy; 7 Epidemiology and Public Health Department of Cardiac Thoracic Vascular Sciences and Public Health, University of Padova, 35128 Padova, Italy; 8 Department of Molecular Medicine (DIMM), University of Padua, 35128 Padua, Italy; 9 General, Administrative and Health Management Unit, Azienda Ospedaliera di Padova, 35128 Padova, Italy

**Keywords:** antimicrobial stewardship, antimicrobial resistance, antimicrobial stewardship program, implementation

## Abstract

**Background/Objectives**: Antimicrobial stewardship programs (ASPs) optimize antimicrobial use, improving outcomes and reducing resistance. This study assessed the impact of a ward-specific ASP. **Methods**: A pre/post quasi-experimental study was conducted in an internal medicine ward at a tertiary hospital in Padua, Italy. During the intervention year (September 2023–August 2024), a multidisciplinary team (infectious disease consultants, pharmacists, microbiologists, nurses, and hygienists) held bi-weekly ward-based audits, reviewing antimicrobial prescriptions and performing bedside assessments. Therapy adjustments followed guidelines and local epidemiology. Educational sessions and infection prevention and control (IPC) protocols were also reinforced. Outcomes were compared to the previous year, considering patient characteristics. The primary outcome was antimicrobial consumption (DDD/100 patient days, DDD/100PD); secondary outcomes included cost savings, length of stay (LOS), and mortality. **Results**: Fifty audits assessed 1074 patients and 1401 antimicrobial treatments. Patient characteristics were similar. Antibiotic suspension or de-escalation occurred in 37.9% and 22% of patients, respectively. AWARE ACCESS class use increased (+17.5%), while carbapenem (−54.4%) and fluoroquinolone (−42.0%) use significantly declined (*p* < 0.05). IPC and microbiological culture guidance were provided in 12.1% of cases. Antimicrobial consumption dropped from 107.7 to 84.4 DDD/100PD (*p* < 0.05). No significant changes in LOS or mortality were observed. Antimicrobial costs fell by 48.8% (with EUR 57,100 saved). **Conclusions**: ASP reduced antimicrobial consumption, improved prescription quality, and cut costs without compromising patient outcomes. Multidisciplinary collaboration, audits, and education proved essential. Future studies should assess long-term resistance trends and integrate rapid diagnostics for enhanced stewardship.

## 1. Introduction

Over the last twenty years, antimicrobial resistance (AMR) has emerged as a pressing global health concern, posing a significant threat to public health and the provision of effective healthcare worldwide; it is a particularly important issue for vulnerable populations [1]. As known, AMR occurs when microorganisms, including bacteria, viruses, fungi, and parasites, evolve to withstand the effects of antimicrobial agents, rendering standard treatments ineffective [2]. The economic burden of AMR on healthcare systems is substantial. Resistant infections necessitate the use of more expensive and toxic second-line or third-line treatments, significantly increasing treatment costs. A systematic review estimated the additional cost of resistant infections to range from USD 6000 to USD 30,000 per patient in high-income countries [3]. As a consequence, the economic burden associated with antimicrobial resistance is also staggering, with the annual global cost estimated to reach USD 100 trillion by 2050 if the current trends continue [4,5].

Moreover, resistant infections often result in extended hospital stays, further straining resources. For instance, studies have shown that methicillin-resistant Staphylococcus aureus (MRSA) infections can prolong hospitalizations by an average of 10 days, amplifying institutional costs [6,7,8]. The magnitude of this crisis cannot be overstated. Indeed, beyond economic implications, AMR profoundly impacts patient mortality and morbidity. According to the World Health Organization (WHO), AMR contributes to an estimated 700,000 deaths annually, which is a figure projected to rise to 10 million by 2050 if current trends persist [1,5,9]. Infections caused by multidrug-resistant organisms (MDROs) are associated with significantly higher mortality rates compared to those caused by susceptible strains. For example, bloodstream infections caused by carbapenem-resistant Enterobacteriaceae (CRE) have a mortality rate exceeding 50% in some cases [10,11].

The rise in AMR is a complex and multifaceted issue driven by a combination of factors, including the inappropriate and excessive use of antimicrobial agents in healthcare settings, agriculture, and the community [12,13]. Addressing this crisis requires a comprehensive, collaborative, and evidence-based approach that involves different levels of healthcare providers, policymakers, researchers, and the public. One of the key strategies in the fight against antimicrobial resistance is the implementation of antimicrobial stewardship programs (ASPs) in healthcare settings, where the spread of MDROs is responsible for a significant burden of morbidity and mortality [14,15].

ASPs are crucial in internal medicine wards, where patients often present with complex comorbidities and prolonged hospital stays, increasing the risk of MDRO infections and inappropriate antimicrobial use [16,17].

These programs aim to optimize the use of antimicrobial agents by promoting appropriate prescribing practices, de-escalating unnecessary use, and monitoring resistance patterns. A meta-analysis demonstrated that ASPs reduce antimicrobial consumption by up to 36%, decrease the incidence of resistant infections, and improve patient outcomes without compromising quality of care [18].

Importantly, ASPs also contribute to cost savings, with institutions reporting reductions in antimicrobial expenditures ranging from 10% to 35% following program implementation [19]. The efficacy of ASPs extends beyond immediate cost reductions and resistance management. These programs foster a culture of accountability and education among healthcare professionals, ensuring that antimicrobial prescribing is evidence-based and aligned with current guidelines [20].

Additionally, ASPs often incorporate diagnostic stewardship to enhance the accuracy of infection diagnoses, further reducing inappropriate antimicrobial use [21].

The aim of this study was to evaluate the impact of a year-long stewardship program, through the adoption of a specific internal medicine department, on antibiotic consumption, patient-related parameters of interest (mortality, length of hospital stay, and Clostridioides difficile disease), and direct costs.

## 2. Results

### 2.1. Patient Profiles and Infection Rates

During the whole period of observation, 3313 patients were discharged from the assessed wards. Details about these patients, both by years of observation and overall, are reported in Table 1. There were no differences between the two groups (no ASP vs. ASP) in terms of sex distribution, age, type of admission, length of stay, the need for ICU admission, weight upon admission, Barthel scale, and type of discharge. Also, considering the prevalence of different infections, there were no significant differences between the two groups, apart from the prevalence of urinary tract infections/pyelonephritis, the prevalence of which was significantly higher during the year with no ASP (Table 2). Overall, the most prevalent infections were pneumonia (17.7%), bloodstream infections (12.5%), and urinary tract infections/pyelonephritis (9.5%).

### 2.2. The Stewardship Program’s Actions and Effects

**Figure 1:**
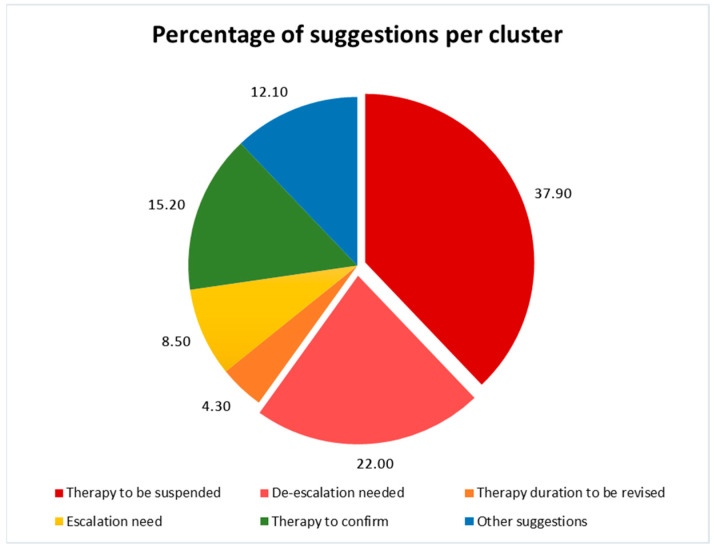
Stewardship suggestions during the ASP intervention phase. Legend for Figure 1. Figure 1 shows the proportion of interventions implemented during the ASP.

During the ASP year, 50 meetings were performed in the selected ward; 1074 patients and 1401 antimicrobial treatments were assessed and reviewed, respectively. The ASP led to the suspension/de-escalation of antibiotics in 37.9% and 22% of cases, respectively. Escalation was required in 8.5% of cases, and a revision of the length of treatment was required in 4.3% of cases. Antibiotic treatment was deemed appropriate and left unchanged in only 15.52% of cases. In 12.1% of cases, other suggestions and advice were provided starting from the clinical case discussion (such as the timing of follow-up blood cultures, the fostering of IPC practice, and the reviewing of treatment guidelines). See Figure 1 for a visual representation of the suggestions provided.

**Figure 2:**
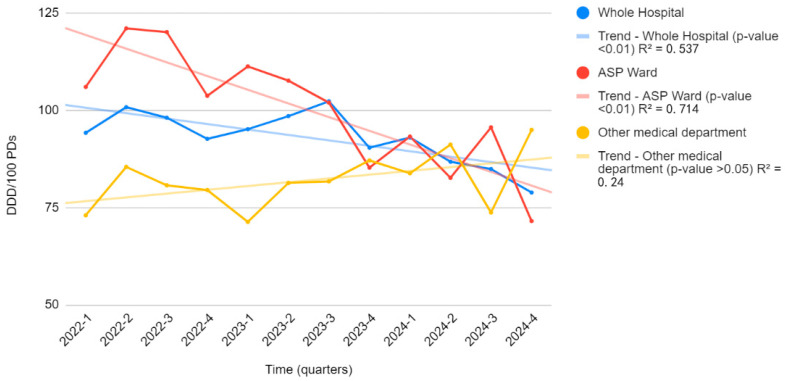
The trend of antimicrobial consumption over time in the ASP ward compared to other medical departments. Legend for Figure 2. Figure 2 shows the trend of antimicrobial consumption (DDD/100 PDs) over time in the ASP ward (red line) compared to other medical departments (yellow line) and the overall hospital consumption (blue line).

Figure 2 illustrates the trend of antimicrobial consumption over time, comparing the ASP ward with other medical departments and the overall hospital. A notable reduction in antimicrobial consumption is observed in the ASP ward following the implementation of the stewardship program, with a significant decrease from 106.0 to 71.5 DDD/100 PDs (*p* = 0.01, R^2^ = 0.715). This strong correlation highlights the effectiveness of ASP interventions in optimizing antimicrobial use over time.

Conversely, in the remaining hospital departments where no ASP was implemented, antimicrobial consumption remained stable with no significant trend (R^2^ = 0.239, *p* > 0.05). Similarly, at the hospital-wide level, although a slight decrease in antimicrobial consumption was observed, the correlation was good and statistically significant (R^2^ = 0.538, *p* < 0.01).

These findings underscore the impact of structured antimicrobial stewardship strategies in reducing unnecessary antimicrobial exposure and promoting more rational prescribing practices while also highlighting the need for broader implementation across all hospital departments to achieve a more substantial reduction in antimicrobial use.

### 2.3. Improvements in Prescription Quality and Costs

We also assessed possible changes in the quality of prescriptions according to the WHO AWaRe classification developed by the World Health Organization. This classification groups antibiotics into three categories: access (first- or second-line antibiotics with lower resistance potential), watch (broader-spectrum agents with higher resistance potential), and reserve (last-resort treatments for multi-resistant infections [22].

**Figure 3:**
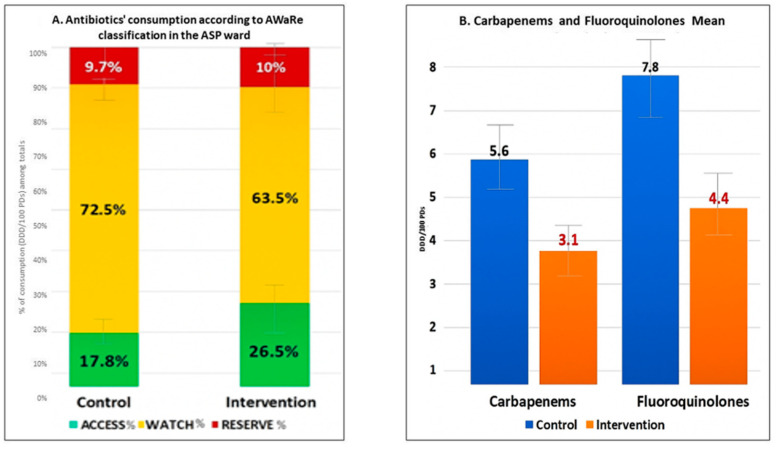
Antibiotic consumption comparison between control and intervention groups, according to WHO-AWARE (Figure 3, **Panel 3A**) and classes of carbapenems and fluoroquinoles (Figure 3, **Panel 3B**).

Notably, we found a significant increase in the use of drugs in the ACCESS class, with the proportion rising from 17.8% at baseline to 26.5% during the intervention period (*p* = 0.04). This suggests a positive shift towards prescribing drugs with a more favorable safety and efficacy profile. In contrast, we observed an important reduction in the use of medications in the WATCH class, from 72.5% in the control group to 63.5% in the intervention group (*p* = 0.04). This decrease reflects a substantial shift away from drugs considered to have a higher risk of adverse effects or limited evidence of efficacy. However, no significant difference was found in the proportion of drugs classified as RESERVE, which remained relatively stable at 9.7% in the control group and 10.0% during the intervention (*p* = 0.41). See Figure 3A for further information. Importantly, we also recorded a significant reduction in both carbapenem (−44.6%) and fluoroquinolone (−43.6%) use (*p* < 0.05 for all), as shown in Figure 3B. These findings indicate that the ASP significantly reduced the use of broad-spectrum antibiotics such as carbapenems and fluoroquinolones, thereby improving the quality of antimicrobial prescribing and potentially enhancing patient safety without adversely affecting clinical outcomes.

No significant changes in major outcomes, such as mortality, length of stay, and mortality, were observed during the two periods, as well as for the prevalence of Clostridioides difficile infections. The overall costs related to antimicrobials in the analyzed unit decreased by 48.8% (EUR 57.100 savings in one year, *p* < 0.01), decreasing from EUR 111,540.00 to EUR 54,440.00 during the ASP year.

### 2.4. Long-Term Impact (Interrupted Time Series Analysis)

**Figure 4:**
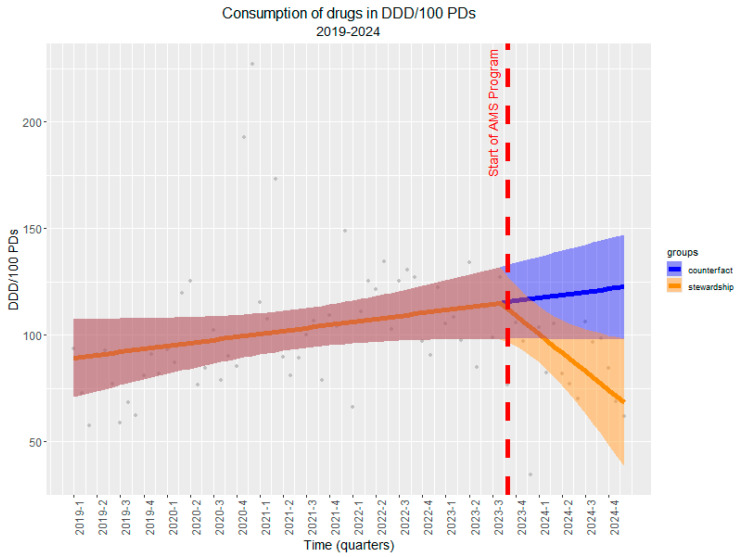
Interrupted time series (ITS) analysis of antibiotic consumption (DDD/100 PDs) before and after the implementation of ASP. Legend for Figure 4. The red dashed line indicates the start of the ASP. The pre-intervention period (1 January 2019–30 September 2023) shows a rising trend in antibiotic use (+0.47 DDD/100 PDs per month), while a significant downward trend (−3.39 DDD/100 PDs per month, *p* = 0.02) is observed post-intervention. The blue line represents the counterfactual scenario where ASP was not introduced, whereas the orange line depicts the observed trend following ASP activation, demonstrating a substantial reduction in antibiotic consumption. Shaded areas indicate 95% confidence intervals.

In order to better contextualize the impact of the ASP in the department, we set up an ITS analysis considering historical antibiotic consumption over the past 5 years (as of January 2019). The analysis demonstrated a significant shift in antibiotic consumption trends following the activation of the program. Prior to the intervention, antibiotic use showed a gradually increasing trend of +0.47 DDD/100 PDs per month. However, after the intervention, there was a marked reversal in this trend, with a significantly reduced trend of −3.39 DDD/100 PDs per month (CI: −6.13, −0.66; *p* = 0.02). This shift is visually evident in Figure 4, where the projected counterfactual trend (in blue) indicates a continued increase in antibiotic use in the absence of the intervention, while the actual observed trend (in orange) shows a sharp and sustained reduction.

**Figure 5:**
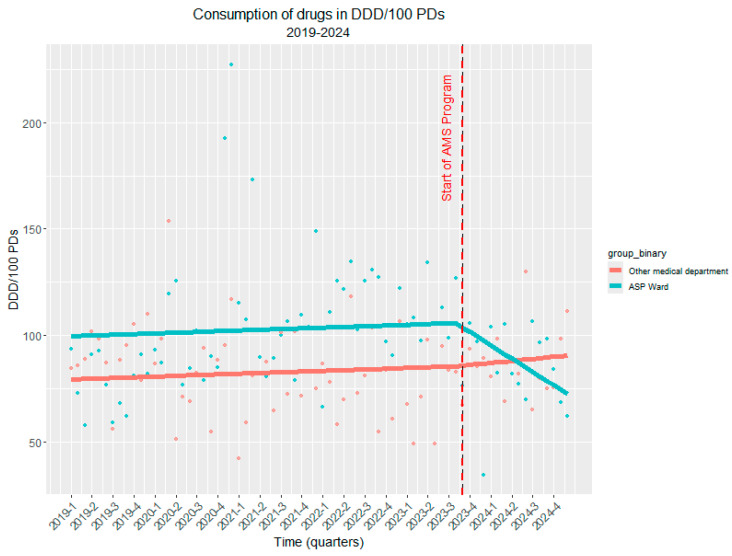
The controlled interrupted time series (ITS) analysis of antibiotic consumption (DDD/100 PDs) before and after the implementation of ASP. Legend for Figure 5. The red dashed line indicates the start of the ASP. The red line represents the consumption of antibiotics in DDD/100 PDs where ASP was not introduced, whereas the blue line depicts the consumption of antibiotics in DDD/100 PDs where ASP was introduced, demonstrating a substantial reduction in antibiotic consumption.

To strengthen causal inference, we also conducted a comparative analysis with a control department that did not receive the ASP intervention during the same period. The comparative analysis using generalized least squares modeling further confirmed these findings. The model revealed that both departments had similar baseline temporal trends (β = 0.11, SE = 0.16 *p* = 0.47). Critically, the interaction term demonstrated that the ASP intervention department experienced a significantly greater reduction in antibiotic use compared to the control department (β = −2.41, SE = 1.15, *p* = 0.038), indicating that the observed decline was specifically attributable to the ASP rather than secular trends or other confounding factors (Figure 5). The comparison with the control department provides additional evidence that this reduction represents a true intervention effect rather than a broader institutional or temporal phenomenon.

**Figure 6:**
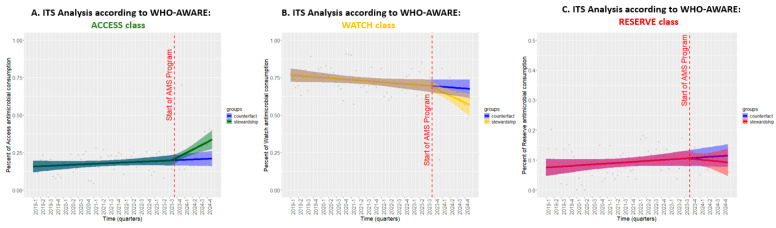
The ITS analysis of antibiotic consumption trends by the AWARE classification before and after the implementation of the ASP. Legend for Figure 6. Each panel represents a different AWARE category: (**A**) ACCESS, (**B**) WATCH, and (**C**) RESERVE. The red dashed line marks the initiation of ASP, and shaded areas represent 95% confidence intervals.

We also investigated the quality of prescriptions by applying the ITS analysis to the assessment of antibiotic consumption stratified by the AWARE classification (Figure 6). For the ACCESS group (Figure 6, Panel A), the pre-intervention period showed a modestly increasing trend of +0.1% per month, which significantly increased by +0.8% per month after ASP activation (CI: 0.2, 1.4; *p* < 0.05), indicating a shift toward the use of antibiotics with a more favorable safety and resistance profile. Conversely, the WATCH category (Figure 6, Panel B) exhibited a declining trend even before ASP implementation (−0.1% per month), but this reduction became substantially steeper post-intervention, reducing by −0.7% per month (CI: −1.4, 0.0; *p* = 0.05), reflecting a targeted effort to limit the use of higher-risk antibiotics. Finally, the use of RESERVE antibiotics (Figure 6, Panel C) remained relatively stable, with a slight pre-intervention increase of +0.1% per month, which transitioned to a minor decrease of −0.1% per quarter after ASP activation (CI: −0.6, 0.3; *p* = 0.51), suggesting no significant impact on this category.

## 3. Discussion

The implementation of a ward-specific ASP in our tertiary-care hospital demonstrated a significant reduction in antimicrobial consumption, improved prescription quality, and substantial cost savings without negatively impacting patient outcomes. These findings align with the growing body of evidence supporting ASPs as a cornerstone in combating AMR [16,17,18,19,20]. Our study identified a 21.7% reduction in antimicrobial consumption (from 106.0107.7 to 71.584.4 DDD/100 PDs), which is a finding comparable to previous research indicating how ASPs can reduce antimicrobial use by 10–36% [23,24]. Additionally, we observed an impressive 48.8% cost reduction associated with antimicrobials, resulting in savings of EUR 57,100 within a single year (*p* < 0.01). Previous studies have similarly demonstrated significant cost savings associated with ASPs, which have been shown to reduce costs by 10–35% [14,25]. This reinforces the issue that these programs not only improve patient safety by minimizing unnecessary antibiotic use but also result in considerable savings for healthcare systems. The financial benefits derived from reduced antibiotic costs present healthcare institutions with an opportunity to reallocate resources more efficiently. For instance, funds saved can be invested in hiring additional infectious disease specialists and nurse epidemiologists. Increasing the presence of these professionals can strengthen the institution’s capacity in infection control, surveillance, and the management of complex cases, ultimately contributing to tackling the challenges posed by infectious diseases.

Importantly, the stable prevalence of Clostridioides difficile infections highlights the need for ongoing vigilance in infection control practices, even as antibiotic usage is optimized. Research indicates a complex relationship between antibiotic stewardship and the incidence of C.difficile infections, suggesting that while reducing broad-spectrum antibiotic use can lower infection risks, other factors such as environmental contamination and patient demographics also play critical roles [26,27].

A key strength of our ASP was its focus on the WHO-AWARE classification. We observed a significant increase in the use of access group antibiotics (+17.5%), a decrease in the use of watch class antibiotics (−12.4%), and substantial reductions in carbapenem (−54.4%) and fluoroquinolone (−42.0%) use. This trend is consistent with global recommendations to prioritize access to antibiotics to mitigate resistance [23]. Similar shifts in antibiotic usage patterns have been reported in ASP evaluations, confirming that targeted interventions successfully drive safer prescribing practices [14,28,29,30].

Unfortunately, the use of the “reserve group” antibiotics did not significantly change across the two evaluation periods. This figure can likely be attributed to the high prevalence of multidrug-resistant organisms in our tertiary care hospital, exacerbated by the admission of complex, colonized patients transferred from other healthcare facilities and long-term care institutions. Notably, in both periods, prescriptions for reserve antibiotics were made only after consultations with infectious disease specialists, ensuring that the prescribing practices were appropriate. Nonetheless, this data underscores the urgent need to implement ASP strategies targeting high-risk antimicrobial use and to enforce infection control measures to mitigate the intra-hospital transmission of multidrug-resistant germs.

The ITS analysis underscored the long-term benefits of ASP implementation. Prior to intervention, antimicrobial consumption exhibited a gradual increase (+0.47 DDD/100 PDs per month), whereas post-ASP, a significant decline (−3.39 DDD/100 PDs per month, *p* = 0.02) was observed. These findings reinforce the sustainability of ASP-driven reductions, comparable to reports from multicenter studies in Europe and North America [31,32].

### Study Limitations and Future Perspectives

Despite these promising results, several challenges remain. Firstly, while our intervention was effective in a single medical ward, broader hospital-wide implementation is essential to achieve more substantial results in reducing and optimizing antibiotic usage. Secondly, ongoing engagement in educational initiatives for all healthcare professionals is crucial, as this will foster a deeper understanding of appropriate antibiotic prescribing practices. Lastly, there must be a commitment to investing in human resources, ensuring that there are enough trained personnel to support these efforts and maintain the effectiveness of the program [32]. Additionally, from a methodological point of view, although we verified the assumptions for segmented linear regression, the potential presence of minor outliers or non-linear trends before the intervention could influence slope estimations. Future studies may benefit from complementary modeling approaches (e.g., Poisson regression or Bayesian ITS). By addressing these areas, we can enhance our approach and maximize stewardship benefits.

Indeed, our multidimensional intervention highlights the importance of multidisciplinary collaboration and consistent monitoring in addressing antimicrobial resistance. By combining targeted audits, education, and stringent IPC protocols, this program sought not only to reduce antimicrobial use but also to enhance patient safety and care quality. The systematic approach ensured that interventions were data-driven and aligned with both institutional goals and global efforts to combat antimicrobial resistance.

## 4. Materials and Methods

This is a pre/post quasi-experimental study assessing the impact of an ASP in a 90-bed internal medicine ward at a tertiary care hospital in Padua, Italy. The study was conducted according to principles of good clinical practice and the Declaration of Helsinki. Patients were requested to sign informed consent for participation, and the study received both a hospital board and Ethics Committee approval (AOP5487). During the intervention year (30 September 2023–31 August 2024), a multidisciplinary team composed of two infectious disease consultants, two hospital pharmacists, two microbiologists, an epidemiologist nurse, and a hygienist conducted bi-weekly audits. These audits involved comprehensive patient assessments, including bedside evaluations and detailed reviews of antimicrobial prescriptions, together with healthcare providers and junior doctors of the selected ward. Based on established guidelines and local epidemiological data, the team implemented measures such as de-escalation, discontinuation, or modification of therapies to optimize antimicrobial use. In addition to the audit meetings, all prescribers had access to institutional and educational programs on antimicrobial use, which were activated prior to the ASP implementation and were not specific to the intervention phase. These initiatives were available across all hospital settings. Moreover, monthly antimicrobial consumption reports were disseminated hospital-wide, including to the ASP ward, as part of routine monitoring.

However, what distinguished the ASP setting was the structured, case-based feedback provided during each bi-weekly audit, where prescriptions were reviewed and discussed directly with the treating team. Targeted educational reinforcements—such as the clarification of guidelines, dosing adjustments, or infection management strategies—were also delivered on a case-by-case basis during these meetings in response to observed prescribing issues or specific clinical challenges. This approach ensured that educational input was practical, timely, and directly linked to patient care. These efforts were designed to enhance awareness and adherence to practices that mitigate the spread of resistant organisms. The ASP’s impact was assessed by comparing data from the intervention year with the previous year (30 September 2022–31 August 2023). This comparison accounted for ward occupancy rates and the demographic and clinical characteristics of patients to ensure accurate and meaningful analysis. Indeed, for each patient admitted over the study period we collected the following information: age, sex, date of birth and discharge, length of stay, death, reasons for admission, coded discharge diagnoses, standard hospital weight, any infectious events (considering viral, bacterial, fungal, and protozoal disease) caused by or occurring during hospitalization per patient, the number of infectious events/patient type of admission (urgent through emergency department or planned), and possible admission to an intensive care unit. We also recorded the Barther Scale at admission. The Barthel Index (also known as the Barthel Scale) is a standardized tool used to assess a patient’s degree of functional independence in performing activities of daily living (ADLs). It is widely used in rehabilitation medicine, geriatrics, neurology, and other clinical settings to evaluate disability, monitor progress during recovery, and inform decisions about care needs and discharge planning. All patients admitted to the ward during the two observation periods were included in the analysis, regardless of whether they received antimicrobial therapy. The primary outcome was total antimicrobial consumption, measured as daily defined doses per 100 patient-days (DDD/100PDs). A linear regression analysis was performed to evaluate the correlation between antimicrobial consumption trends and time across different hospital settings. The coefficient of determination (R^2^) was used to assess the strength of the association, and statistical significance was determined using *p*-values (α = 0.05). Secondary outcomes included cost savings, patient length of stay, and mortality rates. We also considered cases of Clostridioides difficile infection during both periods as a proxy of excess antimicrobial use. These metrics provided a comprehensive evaluation of the program’s effectiveness in improving clinical outcomes and resource utilization. All data collected were anonymized and populated with a Microsoft Excel Spreadsheet. Continuous variables were reported as medians and quartiles (interquartile range—IQR). Between the treatment groups, comparisons were performed for continuous variables via the Mann–Whitney test. Categorical data were expressed as frequency distributions, and Fisher’s exact test was used to determine if differences existed between the groups. We also conducted an interrupted time series (ITS) analysis to evaluate the effectiveness of the ASP implemented in an internal medicine setting in October 2023. The analysis assessed trends in antimicrobial consumption, measured as DDD/100 PDs, over a six-year period from January 2019 to December 2024. Two separate ITS models were performed: one for overall antimicrobial consumption and another stratified according to the WHO AWaRe (access, watch, and reserve) classification. Segmented regression was used to estimate level and trend changes before and after the intervention, accounting for pre-existing trends. Model assumptions—including residual normality, the absence of seasonality, and independence of errors (checked via the Durbin–Watson test)—were verified. We also screened for influential outliers using Cook’s distance and residual diagnostics, confirming the model’s robustness. While generalized or non-linear models were considered, the quarterly nature and distribution of the data supported the use of segmented linear regression. Furthermore, to assess the impact of the ASP intervention, we conducted a controlled, interrupted time series (ITS) analysis comparing antibiotic consumption trends in the ASP ward with those in a non-ASP ward with similar clinical characteristics and mixed cases. Monthly antibiotic use, expressed as defined daily doses per 100 patient-days (DDD/100 PDs), was analyzed for both wards over the period (January 2019–December 2024). The ASP intervention, implemented in September 2023, was treated as an interruption point. This comparative approach allows for better control of secular trends and external factors potentially influencing antimicrobial use. Statistical significance was set at *p* < 0.05. All analyses were performed using R statistical software (version 4.2.3., Vienna, Austria) [32].

## 5. Conclusions

AMR in healthcare settings still continues to represent a significant challenge, with a great burden on mortality rates and increased costs. This high rate of mortality persists even with newer molecules available and is particularly high in patients with advanced age and multimorbidity [33,34,35,36]. The implementation of ASPs, as shown by several experiences, offers a pragmatic and effective solution, combining epidemiological, clinical, economic, and societal benefits [37,38]. However, sustained success requires coordinated efforts across disciplines, robust policy support, and ongoing research to adapt to evolving resistance trends. Addressing AMR is not merely a clinical necessity; it is a critical imperative for global health security.

## Figures and Tables

**Table 1:** Patient features overall and by years of observation (no ASP vs. ASP).

		n Patients per Discharge Period		
Features	Overall n = 3313	2022/2023 (Not ASP Group) n = 1591	2023/2024 (ASP Group) n = 1722	*p*-Value *
**Sex, n (%)**	1572 (47.4)	779 (49)	793 (46.1)	0.094
**Age, years, median (IQR)**	79 (67–86)	79 (67–86)	79 (68–86)	0.371
**Urgent admission, n (%)**	3185 (96.1)	1536 (96.5)	1649 (95.8)	0.243
**Length of stay, days, median (IQR)**	7 (4–12)	7 (5, 12)	7 (4–12)	0.077
**Need of ICU admission, yes, n (%)**	279 (8.4)	143 (9)	136 (7.9)	0.259
**Standard admission weight, median (IQR)**	1.18 (0.91–1.49)	1.15 (0.89–1.49)	1.2 (0.94–1.49)	0.651
**Barthel scale value at admission, median (IQR)**	25 (5–55)	25 (5–55)	25 (5–55)	0.085
**Type of discharge**				0.201
Death, n (%)	330 (10)	158 (9.9)	172 (10)	
Home, n (%)	2035 (61.4)	997 (62.7)	1038 (60.3)	
Home with need of home care, n (%)	496 (15)	236 (14.8)	260 (15.1)	
Nursing home or hospice, n (%)	314 (9.5)	142 (8.9)	172 (10)	
Self-discharge against doctor’s advice, n (%)	37 (1.1)	25 (1.6)	12 (0.7)	
Transferred for care to other hospital, n (%)	25 (0.8)	9 (0.6)	16 (0.9)	
Sent to rehabilitation institutes, n (%)	47 (1.4)	22 (1.4)	25 (1.5)	
Other, n (%)	29 (0.9)	2 (0.1)	27 (1.6)	

* Pearson’s Chi-squared test; Wilcoxon rank sum test; Fisher’s exact test.

**Table 2:** Type and prevalence of different infectious events overall and by years of observation (no ASP vs. ASP).

		n Patients per Discharge Period		
Features	Overall n = 3313	2022/2023 (Not ASP Group) n = 1591	2023/2024 (ASP Group) n = 1722	*p*-Value *
At least one infection, n (%)	1624 (49)	791 (49.7)	833 (48.4)	0.440
At least a bacterial infection, n (%)	1310 (39.5)	644 (40.5)	666 (38.7)	0.289
At least a fungal/protozoal infection, n (%)	21 (0.6)	9 (0.6)	12 (0.7)	0.635
At least a viral (non-COVID-19) infection, n (%)	123 (3.7)	59 (3.7)	64 (3.7)	0.990
COVID-19, n (%)	293 (8.8)	126 (7.9)	167 (9.7)	0.072
Clostridioides difficile, n (%)	26 (0.8)	10 (0.6)	16 (0.9)	0.327
**Type of infection n (%)**				
Pneumonia	586 (17.7)	269 (16.9)	317 (18.4)	0.254
Bloodstream infection	415 (12.5)	198 (12.4)	217 (12.6)	0.892
Septic shock	135 (4.1)	57 (3.6)	78 (4.5)	0.168
Urinary tract infection/pyelonephritis	316 (9.5)	170 (10.7)	146 (8.5)	**0.031**
Septic arthritis	5 (0.2)	5 (0.3)	0 (0)	**0.025**
Endocarditis	15 (0.5)	5 (0.3)	10 (0.6)	0.254
Intra-abdominal infection	84 (2.5)	45 (2.8)	39 (2.3)	0.303
Skin structure and soft tissue infection	87 (2.6)	40 (2.5)	47 (2.7)	0.699
Meningitis	5 (0.2)	4 (0.3)	1 (0.1)	0.201
Prosthetic infection	9 (0.3)	7 (0.4)	2 (0.1)	0.097
Prostatitis	4 (0.1)	2 (0.1)	2 (0.1)	0.99
Osteomyelitis	12 (0.4)	8 (0.5)	4 (0.2)	0.195
Other	147 (4.4)	80 (5)	67 (3.9)	0.112
**N infections/person, n (%)**				0.785
0	1689(51.0)	800 (50.3)	889 (51.6)	
1	944(28.5)	464 (29.2)	480 (27.9)	
2	577 (17.4	282 (17.7)	295 (17.1)	
3	94 (2.8)	41 (2.6)	53 (3.1)	
4	9 (0.3)	4 (0.3)	5 (0.3)	

* Pearson’s Chi-squared test; Fisher’s exact test.

## Data Availability

The data generated herein are available from the corresponding author upon reasonable request.
